# Avian influenza virus prevalence in marine birds is dependent on ocean temperatures

**DOI:** 10.1002/eap.2040

**Published:** 2019-12-27

**Authors:** Jeffrey S. Hall, Robert J. Dusek, Sean W. Nashold, Joshua L. TeSlaa, R. Bradford Allen, Daniel A. Grear

**Affiliations:** ^1^ USGS National Wildlife Health Center 6006 Schroeder Road Madison Wisconsin 53711 USA; ^2^ Maine Department of Inland Fisheries and Wildlife Bangor Maine 04491 USA; ^3^ Present address: Wisconsin Veterinary Diagnostic Laboratory 445 Easterday Lane Madison Wisconsin 53706 USA

**Keywords:** avian influenza, gulls, marine birds, ocean temperature, sea ducks, stability, transmission, virus prevalence

## Abstract

Waterfowl and shorebirds are the primary hosts of influenza A virus (IAV), however, in most surveillance efforts, large populations of birds are not routinely examined; specifically marine ducks and other birds that reside predominately on or near the ocean. We conducted a long‐term study sampling sea ducks and gulls in coastal Maine for IAV and found a virus prevalence (1.7%) much lower than is typically found in freshwater duck populations. We found wide year‐to‐year variation in virus detection in sea ducks and that the ocean water temperature was an important factor affecting IAV prevalence. In particular, the ocean temperature that occurred 11 d prior to collecting virus positive samples was important while water temperature measured concurrently with host sampling had no explanatory power for viral detection. We also experimentally showed that IAV is relatively unstable in sea water at temperatures typically found during our sampling. This represents the first report of virus prevalence and actual environmental data that help explain the variation in marine IAV transmission dynamics.

## Introduction

Environmental factors have large influences on populations and ecosystems. As the world's climate changes, these influences can be drastic and, to a very large extent, the consequences of climate change are unknown. This is particularly true regarding pathogens and disease transmission and prevalence. Any pathogen that is contact, aerosol, water‐borne, or fecally/orally transmitted, necessarily must interact with the physical environment and be able to withstand changes in temperature, humidity, wind, etc. to infect new hosts. Many viral pathogens such as influenza A viruses, are transmitted by these mechanisms and the stability of virions outside of infected hosts is critical for the generation of new infections, viral propagation, as well as the creation of potential human pandemics.

The primary natural reservoir of influenza A viruses (IAV) is wild birds, mainly waterfowl, shorebirds and gulls. These hosts can be infected by all subtypes of IAV with the potential for very rapid genetic reassortment and viral evolution (Webster et al. 1992). This is particularly evident in late summer and autumn when freshwater waterfowl, including large numbers of immunologically naive young of the year, congregate prior to and during their fall migration. The infection rate in these populations can be as high as 70% with distinct risks of infected birds transporting IAV, both short and long distances, in their movements.

Low pathogenic avian influenza viruses (LPAIV) are excreted through the digestive systems of infected wild birds and are spread via a fecal/oral transmission cycle. Whether new hosts become infected by directly contacting virus‐contaminated fecal matter or contaminated water, there are necessarily environmental factors involved in viral persistence and transmission. There have been several laboratory studies that showed a clear influence of water pH, salinity, and temperature on influenza virus stability. LPAIV were most stable in slightly alkaline (pH 7.4–7.8) conditions, freshwater vs. saline water, as well as cooler vs. warmer temperatures (Stallknecht et al. 1990a, b, Brown et al. 2007, 2009). In fact, some viruses retained infectivity after being held >200 d at 4°C (Stallknecht et al. 2010). Low temperatures could provide an important overwintering mechanism for long‐term virus persistence in northern and temperate climates. To date, there have been no studies examining IAV persistence and transmission under actual field conditions.

Marine birds, particularly sea ducks, are traditionally under‐examined in terms of IAV prevalence and their roles in transmission cycles. They are difficult to capture and sample and are most often excluded from surveillance efforts. We conducted influenza virus surveillance in New England marine birds from 2011 to 2013 and 2015 to 2017. Data from these long‐term efforts provided an opportunistic means to examine IAV prevalence and transmission in a marine environment. We modeled how influenza virus detections correspond to local ocean water temperatures, and conducted experimental examination of virus stability in those conditions, with the ultimate goal of understanding the environmental effects on influenza virus persistence and transmission, particularly in light of current climactic change.

## Materials and Methods

### Sea bird sampling

We collected combined oropharyngeal/cloacal swab samples from hunter‐harvested wild sea ducks in various locations along the mid‐coast of Maine during the hunting seasons (November–January) of 2011–2013, 2015–2017. In addition, we opportunistically collected fecal samples from a variety of gull species. The geographic range of sampling locations extended from Frenchman's Bay (44.484031° N, 68.238643° W) in the north to the Kennebec River estuary (43.759961° N, 69.779834° W) in the south. Swab samples were taken using established methods (Hall et al. 2015) and placed in liquid nitrogen vapor shippers for transport to the USGS National Wildlife Health Center and storage at −80°C until analyses.

### RT‐PCR analysis

We extracted viral RNA from swab samples using the Mag‐Max‐96 AI/ND Viral RNA Isolation Kit (Life Technologies, Carlsbad, California, USA) and real‐time polymerase chain reaction (RT‐PCR) analysis performed as described by Spackman et al. 2002. We defined a positive virus detection as amplification within 40 cycles.

### Ocean water temperatures

We obtained ocean temperature data from the National Oceanic and Atmospheric Administration (NOAA; *available online*).^1^ This site provides hourly temperature and physical oceanographic data from a sensor buoy at Bar Harbor, Maine, USA (Station ID 8413320; 44.391630° N, 68.205028° W) and is located within our sampling zone.

### Analysis of ocean temperature and host factor influencing IAV detection

We examined how several host and sampling factors, along with ocean temperature, influenced probability of detecting IAV using a generalized linear regression model with a logit link and binomial error structure for the probability of PCR detection in sample *i*

$$\text{logit}\left(\pi_{i}\right)=\alpha +\beta x+f\left(t\right)$$

In this analysis, **β** is a set of coefficients that describes the effect of a set of predictor variables, **x**, that correspond to each sample. We also considered the effect of ocean temperature, using an optimal temperature dependence function

$$f\left(t\right)=b_{1}*t_{i}+b_{2}*t_{i}^{2}$$

where *t* is the ocean surface temperature metric in association with each sample, *i*. We chose a relatively simple quadratic function to evaluate the effect of ocean temperature because we did not have precise enough measurement of the infection process to warrant a more complex functional form (Low‐Décarie et al. 2017). In addition, the quadratic form is flexible enough such that it can discriminate between an optimal and linear response based on estimating the *b*_1_ and *b*_2_ coefficients.

The set of sample predictor variables, **βx**, were estimated with categorical coefficients in addition to the baseline detection probability, α (regression intercept), and included the effects of categorical covariates of sampling season defined as the year that sampling was initiated during a sampling period, *j* (2011–2013; 2015–2017), the species sampled based on taxonomic order Charadriiformes (gulls) relative to Anseriformes (ducks), and age category of the sampled bird defined as juvenile (hatch year) relative to after‐hatch‐year age

$$\beta x=\beta_{1,j}{\text{season}}_{i}+\beta_{2}{\text{spGull}}_{i}+\beta_{3}\text{age}J_{i}.$$

To examine the effect of ocean temperature we considered potential time lags and different measures of temperature variability to account for the timing of the transmission process and from daily fluctuations in temperature that could influence the environmental component of transmission, respectively. We hypothesized that ocean temperature would not have a direct effect on IAV detection at the same time as sample collection because there are three dynamic transmission components, each with a time period that must be completed sequentially for successful transmission and detection. First, an infected bird must excrete IAV into the environment and that the IAV must remain infective until a susceptible host encounters it. Second, the IAV must cause infection in the new host and undergo an incubation period before it begins to be excreted. Third, excretion persists until the host clears the infection and this step must align with our sampling process. We did not attempt to estimate or examine in detail the dynamics of all these processes; however, we hypothesized that water temperature will have the strongest effect on the initial process when IAV exists free in the environment and longer survival will lead to more transmission events. Hence, we created temperature covariates associated with time lags ranging from 0 (temperature on the day of sampling) to 14 d prior to sampling, as well as for temperature metrics summarized over 0–7, 3–10, and 7–14 d periods prior to sample collection. The choice of daily lag range and weekly periods as covariates was informed by the infectious period of IAV in sea ducks from laboratory infection trials (Hall et al. 2015). In addition to testing for different lag times, we also considered the mean, minimum, and maximum of daily temperature lags and the mean, minimum, maximum, and standard deviation of temperatures aggregated within the 0–7, 3–10, and 7–14 d prior to each sample because temperature variation has been identified as a better descriptor of temperature dependent pathogen dynamics (Paaijmans et al. 2010).

### Model fitting and selection

We used model selection techniques based on Akaike's information criteria (AIC_c_ with correction for sample size) to examine which covariates had the strongest effect on model fit to the observed IAV detection data. We performed model selection in two stages because of the complexity of the temperature lag data. First, we fit a set of candidate models with all additive combinations of the sample covariate data in **βx** and used the AIC difference (ΔAIC_c_) and AIC weights (wAIC_c_) to select the best model (Appendix S1:Table S1; Burnham and Anderson 2002). If there was not a clear best model, we included all covariates included in model sets with ΔAIC_c_ < 4 and also considered the model averaged coefficients to select a parsimonious set of covariates.

Next, we used the parsimonious set of sample covariates selected from **βx** and added the temperature‐dependence function, *f*(*t*), with one temperature‐metric–lag combination at a time to create a new candidate set of models (Appendix S1:Table S1; Appendix S1:Table S2). We chose to fit the temperature function to each time lag and temperature metric combination separately because the metrics and lags were highly correlated. All models were fit with the glm function in R (R Core Team 2017).

### Influenza virus stability under differing environmental conditions

A LPAIV isolate (A/long‐tailed duck/Maine/295/2011 (H3N8)), collected from a sea duck, Long‐tailed Duck (*Clangula hyemalis*), was diluted to 10^5^ EID_50_/mL (where EID50 is the 50% embryo infectious dose) in Instant Ocean sea salt solution prepared according to the manufacturer's instructions (Aquarium Systems, Instant Ocean Spectrum Brands, Blacksburg, Virginia, USA). One‐milliliter aliquots of diluted virus were prepared and stored at −80°C. For each test temperature (4°C, 8°C), we transferred 12 aliquots to a controlled temperature chamber (BTU‐133, Espec, Hudsonville, MI, USA) and held at constant temperature. At weekly intervals (7, 14, 21, and 28 d), three aliquots were removed and stored at −80°C until testing. Additional aliquots were stored at −80°C for the duration of the time course and thawed and frozen one additional time to replicate conditions of the test samples. These served as “day 0” controls. We determined virus titers in 10‐d‐old embryonated chicken eggs (Sunnyside, Beaver Dam, Wisconsin, USA) according to the method of Reed and Muench (1938).

## Results

### Influenza virus prevalence in marine birds

**Figure 1:**
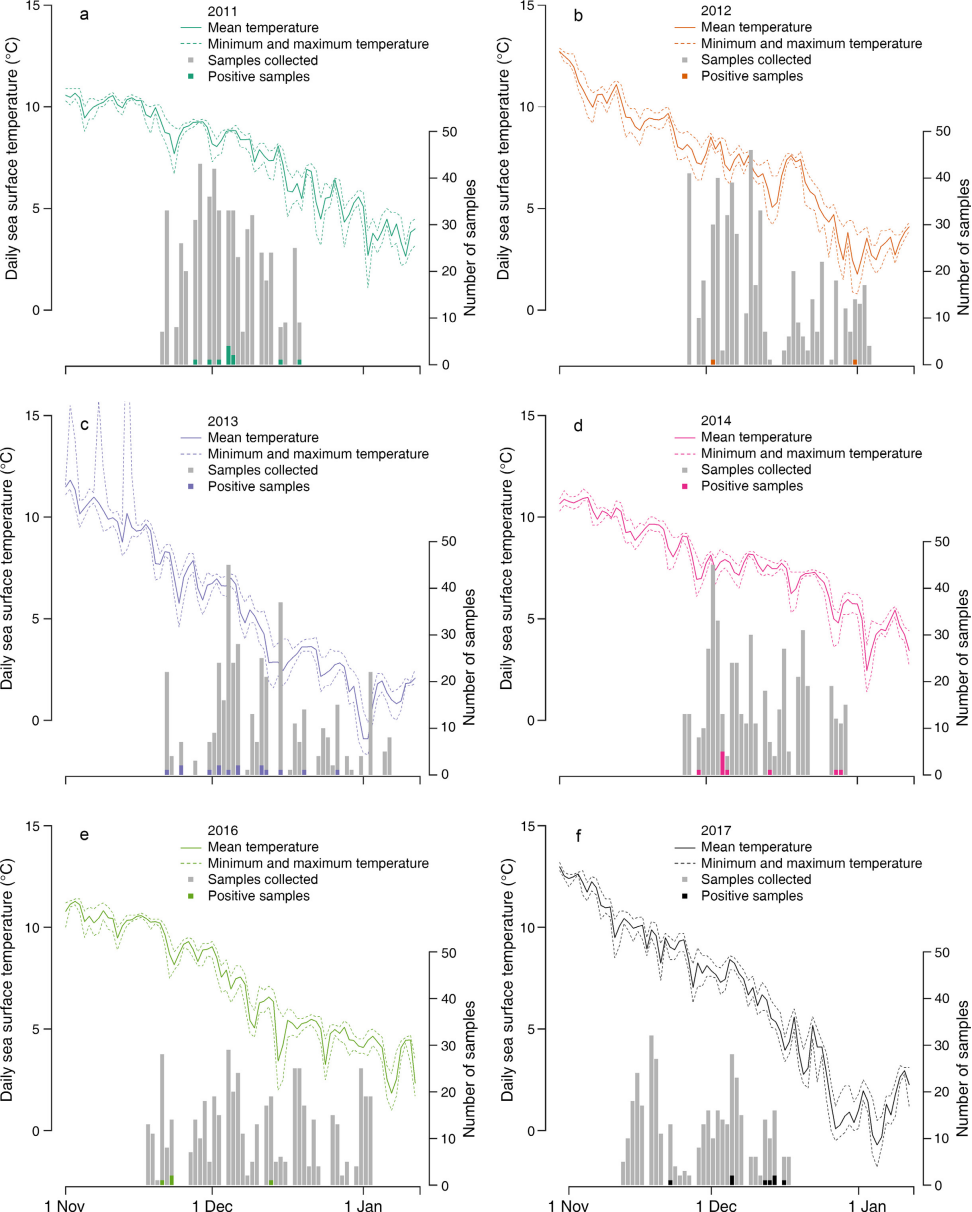
Daily sea temperature mean (solid lines), minimum, and maximum (dashed lines) at the Bar Harbor, Maine, USA (Station ID 8413320) National Oceanic and Atmospheric Administration sensor buoy. Bars represent the number of wild bird samples collected (gray bars) and the number testing positive for influenza A virus (IAV; colored bars) per day, in (a–c) 2011–2013 and (d–f) 2014–2017.

Over the six years of this study, we collected 2,876 swab samples from marine birds. The majority were collected from Common Eiders (*Somateria mollissima*), with lesser numbers taken from Long‐tailed Ducks (*Clangula hyemalis*), three species of Scoters (*Melanitta* sp.), Bufflehead (*Bucephala albeola*), Herring Gull (*Larus argentatus*), Common Goldeneye (*Bucephala clangula*), and miscellaneous other species (Appendix S1:Table S3). We detected a total of 49 IAV by RT‐PCR analysis yielding an overall virus prevalence of 1.7% (49/2,876 total samples). There was year‐to‐year variation in the number of positive samples ranging from only one positive in 2012 to 15 positive samples in 2013 (Fig. 1, Table 1).

**Table 1:** Yearly real‐time polymerase chain reaction (RT‐PCR) detection of influenza A virus infection in wild marine birds

	No. samples		
Year	Collected^a^	RT‐PCR positive^b^	Prevalence (%)
2011	553	11	2.0
2012	532	1	0.2
2013	409	15	3.7
2015	482	10	2.1
2016	510	4	0.8
2017	390	8	2.1
Total	2,876	49	1.7

a Combined oral‐cloacal swabs from hunter harvested birds and fecal samples from gulls collected along the mid‐coast of Maine.

b RT‐PCR cycle threshold (Ct) values of <40.

### Experimental determination of influenza virus stability in sea water at various temperatures

To refine our knowledge regarding ocean temperature and viral detection in sea ducks, we performed controlled experimental examination of virus stability in sea water at temperatures typically encountered in our field surveillance. Viruses in sea water held at constant 4°C lost all ability to infect eggs by 3 weeks and at 8°C, infectivity was completely absent by two weeks (Table 2). Interestingly, while viral infectivity in eggs was completely lost by three weeks at 4°C, the RT‐PCR cycle threshold (Ct) values remained essentially constant over the 4‐week span of the study indicating that the viral RNA remained intact over this time span despite the virus losing infectivity.

**Table 2:** Experimental determination of influenza A virus stability in sea water at constant 4°C and 8°C

Day† and sample	4°C	8°C	4°C RT‐PCR^a^
Day 0			
1	3.31^b^	c	20.72
2	3.30	c	28
3	3.50	c	27.54
Average	3.37 ± 0.09	c	25.42 ± 3.33
Day 7			
1	2.50	1.25	27.66
2	1.75	1.25	27.99
3	2.50	1.60	26.34
Average	2.25 ± 0.35	1.37 ± 0.16	27.33 ± 0.71
Day 14			
1	1.36	>0^d^	28.94
2	1.50	>0^d^	27.54
3	1.28	>0^d^	26.7
Average	1.38 ± 0.09	>0	27.73 ± 0.92
Day 21			
1	>0^d^	>0^d^	26.55
2	>0^d^	>0^d^	27.34
3	>0^d^	>0^d^	26.27
Average	>0	>0	26.72 ± 0.45

Number of days samples held at constant temperature.

a RT‐PCR *C*_*t*_ values of samples held at 4°C.

b log_10_ EID_50_/mL determined in embryonating egg culture.

c Day 0 samples same as 4°C samples.

d No viable virus detected in egg culture.

### Virus detection and ocean water temperature

To determine environmental effects on virus prevalence we charted the daily detection of IAV and the daily local ocean water temperatures for each season of sampling (Fig. 1). The best‐fitting model of the candidate set of sample covariates included only the year effects on IAV detection (wAIC_c_ = 0.49; Table 1; Appendix S1:Table S1). There were three other candidate models with ΔAIC_c_ < 4 and all included the year covariates. We omitted 91 data points that did not have complete data for all covariates (*n* = 2,785). On inspection of relative magnitude and explanatory ability of the fitted coefficients, we chose to only include the year covariate in a parsimonious model because it was the only covariate present in the top models and the only covariate with coefficients that had a significant effect on probability of detection as defined as 95% coefficient confidence intervals not overlapping zero (Table S1).

Among the candidate set of models with the year covariate and sea temperature metric and lag combinations for each sample (*n* = 2,785), the effect of daily minimum sea temperature 11 d prior to the sample collection had the best fit to the data with wAIC_c_ = 0.45 and the next best model with ΔAIC_c_ = 3.8 and wAIC_c_ = 0.07 (Table 3). The coefficient estimates from this model indicated that there was an optimal minimal temperature at the 11‐d lag that explained IAV detection in addition to the effect of year (Table 3; Appendix S1:Table S2).

**Table 3:** Model selection table for candidate models of sample covariates and subsequent set of temperature metric and time lags

Parameters	*k*	log likelihood	AIC_c_	Delta	Weight
Sample covariate models					
Season^a^	6	−233.33	478.69	0	0.49
Season + species^b^	7	−233.09	480.23	1.54	0.23
Season + age^c^	7	−233.33	480.70	2.01	0.18
Season + age + species	8	−233.09	482.23	3.54	0.08
Null (intercept only)	1	−242.50	487.01	8.32	0.01
Species	2	−242.03	488.07	9.38	<0.01
Age	2	−242.50	489.00	10.31	<0.01
Age + species	3	−242.00	490.01	11.33	<0.01
Temperature lag additive models^d^					
Season + *f*(min 11‐d lag)	8	−236.28	488.62	0.00	0.45
Season + *f*(mean 11‐d lag)	8	−238.16	492.38	3.76	0.07
Season + *f*(min 10‐d lag)	8	−238.32	492.69	4.07	0.06
Season + *f*(mean 11‐d lag)	8	−238.50	493.05	4.43	0.05
Season + *f*(min 12‐d lag)	8	−239.06	494.18	5.56	0.03
Season + *f*(max 9‐d lag)	8	−239.49	495.03	6.41	0.02
Season + *f*(min 14‐d lag)	8	−239.61	495.26	6.64	0.02
Season + *f*(min avg. 7–14 d lag)	8	−239.68	495.41	6.79	0.02
Season + *f*(mean 7–14 d lag)	8	−239.77	495.58	6.97	0.01
Season + *f*(mean 9‐d lag)	8	−239.78	495.61	6.99	0.01
Season + *f*(max 4‐d lag)	8	−239.81	495.67	7.05	0.01
Season + *f*(mean 14‐d lag)	8	−239.86	495.76	7.15	0.01
Season + *f*(max 10‐d lag)	8	−239.98	496.02	7.40	0.01
Season + *f*(mean 12‐d lag)	8	−239.99	496.02	7.41	0.01
Season + *f*(min 1‐d lag)	8	−240.00	496.04	7.42	0.01

*Note*

Only the temperature metric and time lag models with wAIC_c_ > 0.01 are shown.

a Season was a categorical variable defined as the year the samples were collected (November–January).

b Species was defined as waterfowl or gull.

c Age was defined as hatch year or after hatch year.

d Optimal temperature functions were added one at a time to the parsimonious sample covariate model.

## Discussion

It is critical to examine virus and other pathogen ecology in relation to their hosts and pathogen–host interactions with the physical environment. This is fundamental to comprehending disease dynamics, spread, and potential risks, and is particularly the case with influenza. In addition, understanding the role of sea ducks and other marine birds in influenza ecology is important. Most of the human population resides near oceans and virus transmission from marine hosts needs to be addressed in surveillance planning, execution, and risk analyses. One important role that marine birds likely play in IAV dynamics is as important species in transporting and/or mixing IAV s inter‐continentally (Ramey et al. 2011, Dusek et al. 2014). In addition, other hosts, particularly marine mammals become infected by avian influenza viruses with periodic mortality events. Recent outbreaks in Northern Europe (Krog et al. 2015) and in the northeastern United States (Anthony et al. 2012) resulted in the deaths of many harbor seals (*Phoca vitulina*) with distinct risks of virus adaptation to mammalian hosts and potential to infect nearby human populations (Karlsson et al. 2014, Guan et al. 2019).

**Figure 2:**
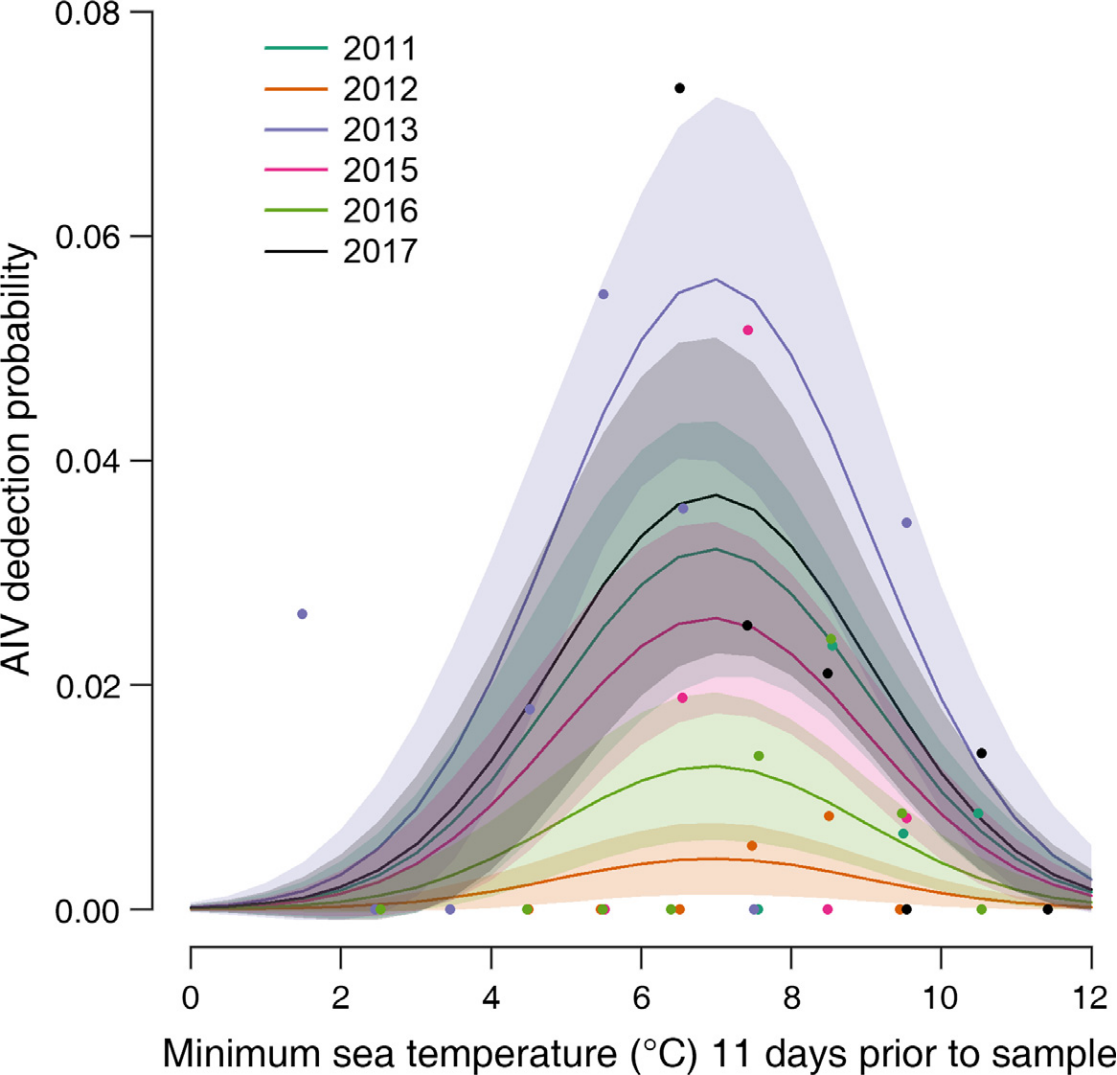
Estimated influenza A virus (IAV) detection probability as a function of sampling year and minimum sea temperature measured 11 d prior to sampling (best fitting model predicted response, mean ± SE; lines and shaded areas) with observed proportion of polymerase chain reaction (PCR) detection aggregated into 2° bins (points).

A fecal/oral pathogen transmission cycle necessarily entails a variety of environmental influences on viral population dynamics. In addition to the yearly variation in sea bird IAV prevalence, our data indicate ocean water temperature plays an important role in IAV transmission, likely by acting on the survival time of virus in the environment. IAV detection in sea ducks predominantly occurred when daily minimum ocean temperatures were between 5°C and 8°C with a detection/sampling time lag of 10–11 d (Fig. 2; Appendix S1: Fig. S1). This finding underscores a frequently overlooked aspect of epidemiology and transmission, that the important environmental conditions existed days or even weeks prior to host sampling and virus detection. In our case, measuring the environmental conditions at the time of sampling, without consideration of the timing and the transmission cycle would have generated a Type II error (not detecting an effect of sea temperature on transmission when there was one). Therefore, defining the ecologically appropriate environmental covariates to measure should incorporate the pathogen transmission cycle (Grear et al. 2013).

Our experimental studies examining the stability of IAV in sea water at relevant temperatures showed that IAV loses infectivity rapidly. After less than three weeks at 4°C, IAV had completely lost the ability to infect chicken eggs, and lost infectivity even quicker at 8°C. These findings contrast with viral stability shown in distilled water where influenza viruses are stable for several months (Stallknecht et al. 2010). These virus stability data, together with the transmission lag time, indicate a relatively narrow window for viral transmission to occur from an infected marine bird to a new host. With ocean temperatures warming, this window likely will get smaller still.

In a previous experimental challenge study, Common Eiders all became infected with LPAIV and cloacally excreted virus in amounts similar to infected Mallards (*Anas platyrhynchos*), a freshwater duck. In addition, water in bathing/swimming tubs provided for the challenged eiders contained significant amounts of IAV, indicating that similar fecal/oral transmission mechanisms are involved in both taxa of ducks (Hall et al. 2015). There are undoubtedly other environmental factors involved in virus stability and transmission in marine ecosystems. Tides, water depth, currents, interactions with biota such as plankton and invertebrates, ultraviolet light, manmade issues such as pollution, etc., potentially could interact with virus populations and have effects on infection and transmission. Other researchers have examined IAV in diving ducks sampled from freshwater environments (Great Lakes, USA) and generally found viral prevalence to be higher than our observed prevalence in marine ducks (Fries et al. 2013, 2014). The reasons for the prevalence difference in freshwater vs. marine ducks are not known; however, our results suggest a compelling hypothesis that ocean temperature and salinity have profound effects on virus stability that could drive IAV transmission and could be the primary reasons for the low IAV prevalence we observe in marine birds.

The interaction of all influenza viruses with the physical environment is a critical component of transmission and persistence and is no less important with IAV infections of sea ducks and other marine hosts. There are very few studies that have investigated environmental factors and influenza in marine habitats and this is the first that we are aware of that integrates actual real time environmental data with virus prevalence in a natural ecosystem. We present our findings in the hope that future studies will help to define the environmental/viral interactions across different host ecologies and aquatic environments.

## Data Availability

Data are available from the Influenza Research Database (https://www.fludb.org/) using the following search criteria: animal surveillance data; surveillance data type, avian; geographic grouping, North America; sampling country, USA; state, Maine; host selection, select species from list: *Somateria mollissima* (Common Eider), *Clangula hyemalis* (Long‐tailed Duck), *Melanitta nigra* (Black Scoter), *Melanitta fusca* (White‐winged Scoter), *Melanitta perspicilata* (Surf Scoter).

## Note

1 https://tidesandcurrents.noaa.gov/physocean.html?id=8413320
