# Supplementary material for: Avian influenza virus prevalence in marine birds is dependent on ocean temperatures
Source: Ecol Appl. 2019 Dec 27;30(2):e02040. doi: 10.1002/eap.2040 (PMC7317457; doi:10.1002/eap.2040)

Supporting Information. Jeffrey S. Hall, Robert J. Dusek, Sean W. Nashold, Joshua L. TeSlaa, R. Bradford Allen, and Daniel A. Grear. 2019. Avian influenza virus prevalence in marine birds is dependent on ocean temperatures. *Ecological Applications.*

Appendix S1

Table S1: Model selection statistics and coefficient estimates for binomial linear models examining the sampling covariates effect on the probability of detection AIV via RT-PCR diagnostics. We excluded 91 swabs for this analysis that did not have data recorded for all covariates (n = 2785). Coefficient estimates and 95% maximum likelihood confidence intervals (CI) are reported on the logit scale. Coefficient with NA values were not included in that candidate model. The set of sample predictor variables, $\boldsymbol{\beta x}$, were estimated with categorical coefficients in addition to the baseline detection probability, α (regression intercept), and included the effects of categorical covariates of sampling season defined as the year that sampling was initiated during a sampling period, j (2012-2013; 2015-2017) relative to 2011, the species sampled based on taxonomic order Charadriiformes (gulls) relative to Anseriformes (ducks), and age category of the sampled bird defined as juvenile (hatch year) relative to adult (after hatch year).

Sample covariate models	k	logLik	AICc	ΔAICc	weight	alpha (intercept)	Age HY	2012	2013	2015	2016	2017	Species Gull
season	6	-233.33	478.69	0	0.49	-3.875	NA	-1.703	0.607	0.027	-0.868	-0.208	NA
						(-4.535, -3.33)		(-3.572, -0.382)	(-0.176, 1.421)	(-0.857, 0.899)	(-2.156, 0.213)	(-1.282, 0.767)	
season + species	7	-233.09	480.23	1.54	0.23	-3.875	NA	-1.729	0.541	-0.007	-0.914	-0.233	0.326
						(-4.535, -3.33)		(-3.6, -0.405)	(-0.272, 1.377)	(-0.897, 0.872)	(-2.21, 0.176)	(-1.31, 0.746)	(-0.673, 1.159)
season + age	7	-233.33	480.7	2.01	0.18	-3.875	-0.011	-1.703	0.607	0.029	-0.866	-0.208	NA
						(-4.536, -3.328)	(-1.236, 0.936)	(-3.572, -0.382)	(-0.177, 1.421)	(-0.87, 0.913)	(-2.162, 0.223)	(-1.282, 0.768)	
season + age + species	8	-233.09	482.23	3.54	0.08	-3.873	-0.058	-1.729	0.541	0.001	-0.907	-0.233	0.332
						(-4.534, -3.327)	(-1.291, 0.90)	(-3.6, -0.405)	(-0.271, 1.377)	(-0.902, 0.89)	(-2.209, 0.19)	(-1.309, 0.746)	(-0.674, 1.174)
null (intercept only)	1	-242.5	487.01	8.32	0.01	-4.043	NA	NA	NA	NA	NA	NA	NA
						(-4.343, -3.771)							
species	2	-242.03	488.07	9.38	<0.01	-4.090	NA	NA	NA	NA	NA	NA	0.452
						(-4.411, -3.799)							(-0.52, 1.243)
age	2	-242.5	489	10.31	<0.01	-4.039	-0.056	NA	NA	NA	NA	NA	NA
						(-4.352, -3.754)	(-1.262, 0.857)						
age + species	3	-242	490.01	11.33	<0.01	-4.081	-0.126	NA	NA	NA	NA	NA	0.468
						(-4.41, -3.782)	(-1.341, 0.8)						(-0.512, 1.269)

Table S2: Model selection statistics and coefficient estimates for binomial linear models examining the season and optimal temperature function w covariates effect on the probability of detection AIV via RT-PCR diagnostics (n = 2876). We performed model selection by comparing models with one sea temperature metric-time lag combination at a time because of the high co-linearity of the sea temperature data. Our candidate set of models included daily mean, daily minimum, and daily maximum sea temperature at daily time lags of 0 (temperature on the day of sampling) to 14 days prior to sampling and with the mean, minimum, maximum, and standard deviation of temperatures aggregated within the 0-7, 3-10, and 7-14 days prior to each sample. The effects of categorical covariates of sampling season defined as the year that sampling was initiated during a sampling period, *j* (2012-2013; 2015-2017) relative to 2011. Coefficient estimates and 95% maximum likelihood confidence intervals (CI) are reported on the logit scale.

													Optimal temperature function coefficeints	
Days lag	sea temp metric	k	logLik	AICc	ΔAICc	weight	alpha (intercept)	Season 2012	Season 2013	Season 2015	Season 2016	Season 2017	t	t^2
11	Min	8	-236.28	488.62	0.00	0.45	-9.25	-1.99	0.59	-0.22	-0.94	0.14	1.68	-0.12
							(-15.44, -5.03)	(-3.88, -0.63)	(-0.36, 1.54)	(-1.12, 0.68)	(-2.26, 0.17)	(-0.82, 1.06)	(0.57, 3.31)	(-0.23, -0.05)
11	Mean	8	-238.16	492.38	3.76	0.07	-10.32	-1.81	0.79	-0.10	-0.81	0.23	1.70	-0.11
							(-17.95, -5.27)	(-3.7, -0.46)	(-0.14, 1.73)	(-1, 0.8)	(-2.12, 0.29)	(-0.74, 1.15)	(0.47, 3.53)	(-0.22, -0.03)
10	Min	8	-238.32	492.69	4.07	0.06	-8.93	-1.72	0.90	-0.05	-0.76	0.15	1.40	-0.09
							(-14.69, -5.02)	(-3.6, -0.36)	(-0.02, 1.83)	(-0.96, 0.85)	(-2.07, 0.35)	(-0.81, 1.07)	(0.37, 2.9)	(-0.19, -0.02)
10	Mean	8	-238.50	493.05	4.43	0.05	-10.07	-1.73	0.87	-0.05	-0.76	0.20	1.61	-0.10
							(-17.49, -5.23)	(-3.62, -0.38)	(-0.07, 1.8)	(-0.97, 0.85)	(-2.07, 0.36)	(-0.77, 1.11)	(0.4, 3.43)	(-0.22, -0.02)
12	Min	8	-239.06	494.18	5.56	0.03	-7.15	-1.94	0.52	-0.19	-0.99	0.17	1.08	-0.08
							(-11.79, -3.72)	(-3.83, -0.58)	(-0.41, 1.46)	(-1.1, 0.71)	(-2.3, 0.13)	(-0.8, 1.08)	(0.18, 2.3)	(-0.16, -0.02)
9	Max	8	-239.49	495.03	6.41	0.02	-9.98	-1.71	0.83	-0.03	-0.78	0.18	1.49	-0.09
							(-18.11, -4.72)	(-3.59, -0.36)	(-0.1, 1.77)	(-0.95, 0.87)	(-2.1, 0.33)	(-0.78, 1.1)	(0.23, 3.39)	(-0.2, -0.01)
14	Min	8	-239.61	495.26	6.64	0.02	-9.13	-1.65	0.89	0.04	-0.77	0.24	1.27	-0.08
							(-15.98, -4.57)	(-3.53, -0.3)	(0.02, 1.78)	(-0.87, 0.94)	(-2.07, 0.33)	(-0.73, 1.17)	(0.18, 2.89)	(-0.17, -0.01)
7-14	Min	8	-239.68	495.41	6.79	0.02	-7.01	-1.64	1.05	-0.01	-0.68	0.10	0.95	-0.07
							(-10.88, -4.21)	(-3.52, -0.29)	(0.07, 2.03)	(-0.91, 0.89)	(-1.99, 0.42)	(-0.86, 1.02)	(0.13, 2.08)	(-0.16, -0.01)
7-14	Mean	8	-239.77	495.58	6.97	0.01	-9.10	-1.70	0.88	-0.02	-0.77	0.19	1.30	-0.08
							(-16.2, -4.48)	(-3.59, -0.34)	(-0.07, 1.83)	(-0.94, 0.89)	(-2.08, 0.34)	(-0.78, 1.11)	(0.17, 2.99)	(-0.18, -0.01)
9	Mean	8	-239.78	495.61	6.99	0.01	-8.62	-1.70	0.82	-0.04	-0.79	0.13	1.23	-0.08
							(-15.1, -4.41)	(-3.59, -0.35)	(-0.11, 1.76)	(-0.97, 0.87)	(-2.1, 0.33)	(-0.83, 1.05)	(0.15, 2.83)	(-0.18, -0.01)
4	Max	8	-239.81	495.67	7.05	0.01	-7.68	-1.86	0.56	-0.18	-1.00	0.09	1.18	-0.08
							(-13.12, -3.75)	(-3.75, -0.49)	(-0.44, 1.55)	(-1.11, 0.73)	(-2.35, 0.14)	(-0.87, 1.01)	(0.13, 2.6)	(-0.18, -0.01)
14	Mean	8	-239.86	495.76	7.15	0.01	-9.74	-1.62	0.92	0.07	-0.77	0.25	1.33	-0.07
							(-17.7, -4.53)	(-3.5, -0.28)	(0.05, 1.81)	(-0.84, 0.96)	(-2.07, 0.32)	(-0.73, 1.18)	(0.14, 3.1)	(-0.18, 0)
10	Max	8	-239.98	496.02	7.40	0.01	-9.69	-1.62	1.02	0.07	-0.74	0.17	1.28	-0.07
							(-17.49, -4.45)	(-3.5, -0.28)	(0.1, 1.94)	(-0.84, 0.96)	(-2.04, 0.36)	(-0.81, 1.1)	(0.08, 3.03)	(-0.17, 0)
12	Mean	8	-239.99	496.02	7.41	0.01	-7.94	-1.83	0.66	-0.11	-0.90	0.21	1.13	-0.07

							(-13.66, -3.82)	(-3.71, -0.47)	(-0.28, 1.6)	(-1.03, 0.79)	(-2.22, 0.2)	(-0.76, 1.12)	(0.11, 2.52)	(-0.16, -0.01)
1	Min	8	-240.00	496.04	7.42	0.01	-5.13	-1.89	0.49	-0.21	-1.10	0.02	0.62	-0.06
							(-7.49, -3.27)	(-3.78, -0.53)	(-0.45, 1.45)	(-1.13, 0.71)	(-2.44, 0.04)	(-0.94, 0.94)	(0.04, 1.38)	(-0.12, -0.01)
13	Mean	8	-240.16	496.37	7.75	0.01	-8.30	-1.75	0.74	-0.05	-0.86	0.22	1.14	-0.07
							(-14.74, -3.93)	(-3.63, -0.4)	(-0.16, 1.65)	(-0.96, 0.85)	(-2.16, 0.25)	(-0.75, 1.14)	(0.09, 2.65)	(-0.16, -0.01)
14	Max	8	-240.19	496.43	7.81	0.01	-9.09	-1.56	1.10	0.16	-0.77	0.10	0.99	-0.05
							(-15.9, -4.23)	(-3.43, -0.23)	(0.21, 2)	(-0.74, 1.05)	(-2.07, 0.32)	(-0.88, 1.04)	(0.06, 2.34)	(-0.12, 0)
9	Min	8	-240.22	496.49	7.87	0.01	-7.38	-1.64	0.91	0.02	-0.77	0.09	0.90	-0.06
							(-12.2, -4.07)	(-3.52, -0.29)	(-0.02, 1.86)	(-0.91, 0.93)	(-2.08, 0.34)	(-0.87, 1.01)	(0.02, 2.17)	(-0.14, 0.01)
12	Max	8	-240.27	496.59	7.97	0.01	-9.34	-1.75	0.77	-0.04	-0.84	0.24	1.33	-0.08
							(-16.9, -3.98)	(-3.63, -0.4)	(-0.16, 1.69)	(-0.95, 0.86)	(-2.15, 0.26)	(-0.73, 1.17)	(0.08, 3.04)	(-0.18, 0)
1	Mean	8	-240.31	496.66	8.04	0.01	-5.78	-1.87	0.54	-0.19	-1.04	0.06	0.74	-0.06
							(-8.95, -3.41)	(-3.76, -0.5)	(-0.43, 1.53)	(-1.12, 0.74)	(-2.39, 0.1)	(-0.9, 0.97)	(0.03, 1.7)	(-0.14, 0)
5	Max	8	-240.38	496.80	8.18	0.01	-8.10	-1.75	0.76	-0.08	-0.89	0.11	1.15	-0.08
							(-13.99, -3.89)	(-3.64, -0.39)	(-0.21, 1.73)	(-1.01, 0.83)	(-2.2, 0.23)	(-0.85, 1.03)	(0.04, 2.63)	(-0.17, 0)
3-10	Mean	8	-240.42	496.90	8.28	0.01	-7.50	-1.73	0.82	-0.06	-0.82	0.11	0.99	-0.07
							(-12.72, -3.88)	(-3.62, -0.35)	(-0.17, 1.8)	(-0.99, 0.86)	(-2.15, 0.3)	(-0.86, 1.02)	(0.02, 2.35)	(-0.16, 0)
3-10	Min	8	-240.43	496.91	8.29	0.01	-7.03	-1.78	0.69	-0.07	-0.90	0.17	0.89	-0.06
							(-12.06, -3.59)	(-3.66, -0.43)	(-0.22, 1.61)	(-0.99, 0.83)	(-2.21, 0.21)	(-0.79, 1.09)	(0.02, 2.15)	(-0.14, 0)
1	Max	8	-240.44	496.93	8.32	0.01	-6.63	-1.84	0.59	-0.17	-1.00	0.10	0.91	-0.07
							(-10.71, -3.56)	(-3.73, -0.47)	(-0.4, 1.59)	(-1.11, 0.76)	(-2.35, 0.14)	(-0.86, 1.01)	(0.02, 2.07)	(-0.15, 0)
13	Max	8	-240.44	496.93	8.32	0.01	-9.13	-1.67	0.88	0.03	-0.80	0.23	1.19	-0.07
							(-16.58, -3.92)	(-3.55, -0.34)	(-0.01, 1.78)	(-0.87, 0.92)	(-2.11, 0.29)	(-0.75, 1.16)	(0.03, 2.8)	(-0.16, 0)
8	Mean	8	-240.46	496.98	8.36	0.01	-7.44	-1.58	1.04	0.09	-0.75	0.08	0.80	-0.04
							(-12.64, -3.8)	(-3.46, -0.23)	(0.12, 1.96)	(-0.83, 1)	(-2.05, 0.35)	(-0.88, 1.01)	(-0.14, 2.08)	(-0.13, 0.02)
5	Mean	8	-240.48	497.00	8.38	0.01	-6.70	-1.81	0.66	-0.11	-0.94	0.06	0.89	-0.06
							(-11.15, -3.52)	(-3.71, -0.44)	(-0.32, 1.64)	(-1.03, 0.79)	(-2.26, 0.19)	(-0.9, 0.98)	(0.02, 2.08)	(-0.15, 0)
NA	none	6	-242.49	497.01	8.39	0.01	-3.90	-1.68	0.63	0.04	-0.94	0.03	NA	NA
							(-4.56, -3.35)	(-3.55, -0.36)	(-0.15, 1.44)	(-0.84, 0.92)	(-2.23, 0.14)	(-0.93, 0.95)		
4	Mean	8	-240.49	497.04	8.42	0.01	-6.07	-1.83	0.59	-0.15	-0.97	0.04	0.77	-0.06
							(-9.89, -3.33)	(-3.73, -0.46)	(-0.41, 1.58)	(-1.07, 0.77)	(-2.31, 0.17)	(-0.92, 0.96)	(0, 1.83)	(-0.14, 0)
8	Min	8	-240.51	497.07	8.45	0.01	-6.42	-1.51	1.11	0.16	-0.74	0.04	0.52	-0.03
							(-10.28, -3.62)	(-3.39, -0.15)	(0.18, 2.06)	(-0.77, 1.07)	(-2.04, 0.36)	(-0.92, 0.96)	(-0.24, 1.53)	(-0.1, 0.03)
11	Max	8	-240.58	497.21	8.59	0.01	-4.61	-1.57	0.80	0.13	-0.85	-0.06	0.03	0.00
							(-8.06, -1.52)	(-3.45, -0.24)	(-0.18, 1.75)	(-0.76, 1.02)	(-2.15, 0.24)	(-1.02, 0.86)	(-0.47, 0.6)	(-0.02, 0.03)
7	Max	8	-240.60	497.24	8.63	0.01	-8.24	-1.58	1.06	0.09	-0.75	0.10	0.95	-0.05
							(-14.4, -3.72)	(-3.46, -0.24)	(0.14, 1.98)	(-0.82, 0.98)	(-2.05, 0.35)	(-0.87, 1.02)	(-0.12, 2.36)	(-0.14, 0.01)
4	Min	8	-240.74	497.53	8.92	0.01	-5.53	-1.78	0.67	-0.11	-0.91	0.03	0.61	-0.05
							(-8.55, -3.32)	(-3.67, -0.41)	(-0.31, 1.64)	(-1.03, 0.8)	(-2.24, 0.22)	(-0.94, 0.94)	(-0.03, 1.48)	(-0.12, 0.0)
0-7	Max	8	-240.77	497.59	8.97	0.01	-8.25	-1.64	0.96	0.04	-0.79	0.11	0.99	-0.06
							(-14.64, -3.63)	(-3.52, -0.29)	(0.03, 1.88)	(-0.87, 0.94)	(-2.1, 0.31)	(-0.85, 1.03)	(-0.11, 2.46)	(-0.14, 0.01)
3-10	Min	8	-240.78	497.60	8.99	0.01	-5.53	-1.69	0.79	-0.06	-0.73	0.06	0.63	-0.06

							(-8.24, -3.47)	(-3.58, -0.33)	(-0.22, 1.78)	(-0.97, 0.85)	(-2.07, 0.4)	(-0.9, 0.97)	(-0.03, 1.52)	(-0.14, 0.01)
3	Max	8	-240.82	497.70	9.08	0.00	-6.81	-1.82	0.62	-0.14	-0.96	0.08	0.91	-0.07
							(-11.76, -3.28)	(-3.71, -0.45)	(-0.4, 1.62)	(-1.08, 0.79)	(-2.31, 0.18)	(-0.88, 0.99)	(-0.06, 2.22)	(-0.15, 0.01)
7	Mean	8	-240.85	497.75	9.13	0.00	-6.61	-1.53	1.05	0.12	-0.75	0.06	0.57	-0.03
							(-11.04, -3.34)	(-3.42, -0.17)	(0.12, 1.98)	(-0.8, 1.03)	(-2.05, 0.34)	(-0.91, 0.98)	(-0.31, 1.69)	(-0.1, 0.03)
0	Max	8	-240.87	497.78	9.16	0.00	-6.06	-1.84	0.59	-0.13	-1.01	0.07	0.75	-0.06
							(-9.94, -3.29)	(-3.73, -0.46)	(-0.4, 1.57)	(-1.07, 0.79)	(-2.36, 0.12)	(-0.9, 0.98)	(-0.06, 1.88)	(-0.14, 0.01)
0-7	Mean	8	-240.90	497.85	9.23	0.00	-6.34	-1.78	0.70	-0.10	-0.91	0.07	0.77	-0.06
							(-10.3, -3.44)	(-3.68, -0.4)	(-0.31, 1.71)	(-1.05, 0.83)	(-2.25, 0.22)	(-0.9, 0.98)	(-0.07, 1.88)	(-0.14, 0.01)
7	Min	8	-240.93	497.90	9.28	0.00	-5.95	-1.50	1.07	0.13	-0.75	0.05	0.42	-0.02
							(-9.35, -3.37)	(-3.39, -0.14)	(0.14, 2)	(-0.78, 1.04)	(-2.05, 0.35)	(-0.92, 0.97)	(-0.3, 1.33)	(-0.08, 0.03)
0-7	Min	8	-240.97	497.98	9.36	0.00	-4.92	-1.73	0.72	-0.06	-0.83	0.06	0.45	-0.04
							(-6.96, -3.31)	(-3.62, -0.36)	(-0.26, 1.7)	(-0.99, 0.85)	(-2.16, 0.29)	(-0.9, 0.97)	(-0.05, 1.15)	(-0.11, 0.01)
0-7	SD	8	-240.97	497.98	9.37	0.00	-6.00	-1.79	0.60	0.01	-0.85	-0.09	6.30	-4.22
							(-9.02, -3.53)	(-3.67, -0.44)	(-0.25, 1.47)	(-0.89, 0.89)	(-2.16, 0.26)	(-1.06, 0.84)	(-0.74, 15.07)	(-10.4, 0.48)
6	Max	8	-240.97	498.00	9.38	0.00	-7.70	-1.69	0.83	-0.01	-0.84	0.11	0.96	-0.06
							(-13.67, -3.44)	(-3.58, -0.33)	(-0.13, 1.78)	(-0.93, 0.9)	(-2.15, 0.27)	(-0.85, 1.03)	(-0.15, 2.43)	(-0.15, 0.02)
2	Max	8	-240.99	498.03	9.41	0.00	-6.79	-1.77	0.69	-0.11	-0.92	0.08	0.87	-0.06
							(-11.33, -3.36)	(-3.67, -0.41)	(-0.31, 1.69)	(-1.06, 0.83)	(-2.26, 0.21)	(-0.88, 1)	(-0.11, 2.11)	(-0.15, 0.01)
6	Mean	8	-241.12	498.28	9.66	0.00	-6.69	-1.68	0.83	-0.01	-0.85	0.08	0.75	-0.05
							(-11.34, -3.39)	(-3.58, -0.31)	(-0.14, 1.79)	(-0.93, 0.89)	(-2.17, 0.26)	(-0.88, 0.99)	(-0.15, 1.96)	(-0.13, 0.02)
0	Mean	8	-241.35	498.75	10.13	0.00	-4.95	-1.84	0.54	-0.12	-1.05	0.02	0.47	-0.04
							(-7.65, -2.94)	(-3.73, -0.46)	(-0.44, 1.53)	(-1.05, 0.8)	(-2.39, 0.09)	(-0.94, 0.94)	(-0.13, 1.31)	(-0.11, 0.01)
3-10	Max	8	-241.36	498.77	10.16	0.00	-6.39	-1.55	1.05	0.15	-0.79	-0.01	0.41	-0.02
							(-10.23, -3.12)	(-3.43, -0.22)	(0.1, 1.99)	(-0.75, 1.03)	(-2.09, 0.3)	(-0.97, 0.91)	(-0.13, 1.04)	(-0.04, 0.01)
3	Mean	8	-241.38	498.81	10.19	0.00	-5.51	-1.80	0.62	-0.12	-0.95	0.03	0.57	-0.05
							(-8.84, -2.99)	(-3.7, -0.42)	(-0.4, 1.62)	(-1.07, 0.81)	(-2.3, 0.19)	(-0.94, 0.95)	(-0.16, 1.53)	(-0.12, 0.01)
7-14	Max	8	-241.40	498.86	10.24	0.00	-5.42	-1.59	0.81	0.13	-0.85	-0.05	0.20	-0.01
							(-9.48, -1.93)	(-3.47, -0.26)	(-0.17, 1.76)	(-0.77, 1.01)	(-2.15, 0.24)	(-1.01, 0.87)	(-0.35, 0.83)	(-0.03, 0.02)
2	Mean	8	-241.43	498.91	10.29	0.00	-5.46	-1.74	0.72	-0.07	-0.92	0.04	0.50	-0.04
							(-8.38, -3.19)	(-3.64, -0.36)	(-0.29, 1.74)	(-1.02, 0.88)	(-2.25, 0.21)	(-0.92, 0.96)	(-0.17, 1.36)	(-0.1, 0.02)
5	Min	8	-241.43	498.91	10.29	0.00	-5.34	-1.75	0.67	-0.06	-0.93	0.03	0.50	-0.04
							(-8.59, -3.04)	(-3.64, -0.38)	(-0.3, 1.65)	(-0.96, 0.84)	(-2.25, 0.19)	(-0.93, 0.95)	(-0.14, 1.41)	(-0.11, 0.01)
6	Min	8	-241.56	499.18	10.56	0.00	-5.64	-1.62	0.88	0.03	-0.83	0.05	0.45	-0.03
							(-9.09, -3.18)	(-3.52, -0.24)	(-0.1, 1.85)	(-0.88, 0.94)	(-2.14, 0.28)	(-0.91, 0.97)	(-0.23, 1.4)	(-0.1, 0.03)
8	Max	8	-241.57	499.19	10.57	0.00	-5.98	-1.55	1.02	0.15	-0.79	0.00	0.35	-0.01
							(-9.46, -2.95)	(-3.42, -0.21)	(0.06, 1.98)	(-0.75, 1.04)	(-2.09, 0.3)	(-0.96, 0.92)	(-0.16, 0.94)	(-0.04, 0.01)
2	Min	8	-241.60	499.25	10.63	0.00	-4.95	-1.70	0.76	-0.02	-0.89	0.04	0.35	-0.03
							(-7.16, -3.2)	(-3.6, -0.32)	(-0.23, 1.76)	(-0.97, 0.92)	(-2.22, 0.23)	(-0.93, 0.95)	(-0.16, 1.01)	(-0.08, 0.02)
3	Min	8	-241.65	499.35	10.73	0.00	-4.87	-1.78	0.61	-0.10	-0.94	0.01	0.40	-0.03
							(-7.35, -2.91)	(-3.68, -0.4)	(-0.4, 1.61)	(-1.05, 0.84)	(-2.29, 0.2)	(-0.96, 0.93)	(-0.18, 1.16)	(-0.1, 0.02)
0	Min	8	-241.89	499.82	11.21	0.00	-4.30	-1.79	0.55	-0.07	-1.04	0.01	0.25	-0.03

							(-6.24, -2.75)	(-3.69, -0.42)	(-0.43, 1.53)	(-0.98, 0.84)	(-2.38, 0.09)	(-0.95, 0.93)	(-0.19, 0.87)	(-0.08, 0.02)
7-14	SD	8	-242.18	500.41	11.79	0.00	-4.78	-1.80	0.49	0.01	-0.99	-0.09	2.31	-1.23
							(-7.56, -2.43)	(-3.7, -0.43)	(-0.43, 1.41)	(-0.88, 0.89)	(-2.32, 0.13)	(-1.09, 0.87)	(-4.01, 9.94)	(-6.18, 2.64)
7-14	SD	8	-242.39	500.83	12.22	0.00	-3.72	-1.62	0.74	0.07	-0.89	0.09	-0.35	0.02
							(-6.18, -1.66)	(-3.51, -0.27)	(-0.19, 1.69)	(-0.83, 0.96)	(-2.2, 0.22)	(-0.92, 1.06)	(-6.48, 6.72)	(-4.77, 4.09)

Table S3. Numbers of each species sampled in each year of the study and number of influenza virus detections for each species.

Species	2011	2012	2013	2015	2016	2017	Total	PCR Positive
Barrow's Goldeneye, *Bucephala islandica*	1			1			2	
Black Duck, *Anas rubripes*	6	13	11	3	2	4	39	
Black Scoter, *Melanitta nigra*	5	27	7	14	8	21	82	1
Bufflehead, *Bucephala albeola*	114	36	47	50	60	37	344	12
Canada Goose, *Branta canadensis*					2	2	4	
Common Eider, *Somateria mollissima*	238	256	134	189	246	221	1284	13
Common Goldeneye, *Bucephala clangula*	16	18	26	37	10	6	113	2
Great Black-backed Gull, *Larus marinus*		1				1	2	
Greater Scaup, *Aythya marila*				3		1	4	
Herring Gull, *Larus argentatus*	5	32	67	38	91	43	276	6
Hooded Merganser, *Lophodytes cucullatus*				1				
King Eider, *Somateria spectabilis*						1	1	
Lesser Scaup, *Aythya affinis*					1		1	
Long-tailed Duck, *Clangula hyemalis*	113	67	61	96	38	29	404	8
Mallard, *Anas platyrhynchos*	2	8	9	2	13	2	36	2
Northern Shoveler, *Anas clypeata*			1				1	1
Ring-billed Gull, *Larus delawarensis*	7	4	6	5	2	2	26	
Red-breasted Merganser, *Mergus serrator*	20	15	6	5	7	9	62	
Ruddy Duck, *Oxyura jamaicensis*		1					1	
Surf Scoter, *Melanitta perspicillata*	10	7	13	29	13	3	75	2
White-winged Scoter, *Histrionicus histrionicus*	17	47	21	9	17	8	119	2
Total	554	532	409	482	510	390	2876	49

Figure S1. Model selection using AIC weights (wAIC) on avian influenza virus detection as the response to optimal sea temperature function across 0-14 days prior to sampling (lag) and temperature metric indicated the best fitting model included the effect of sampling year and the minimum temperature 11 days prior to sampling.

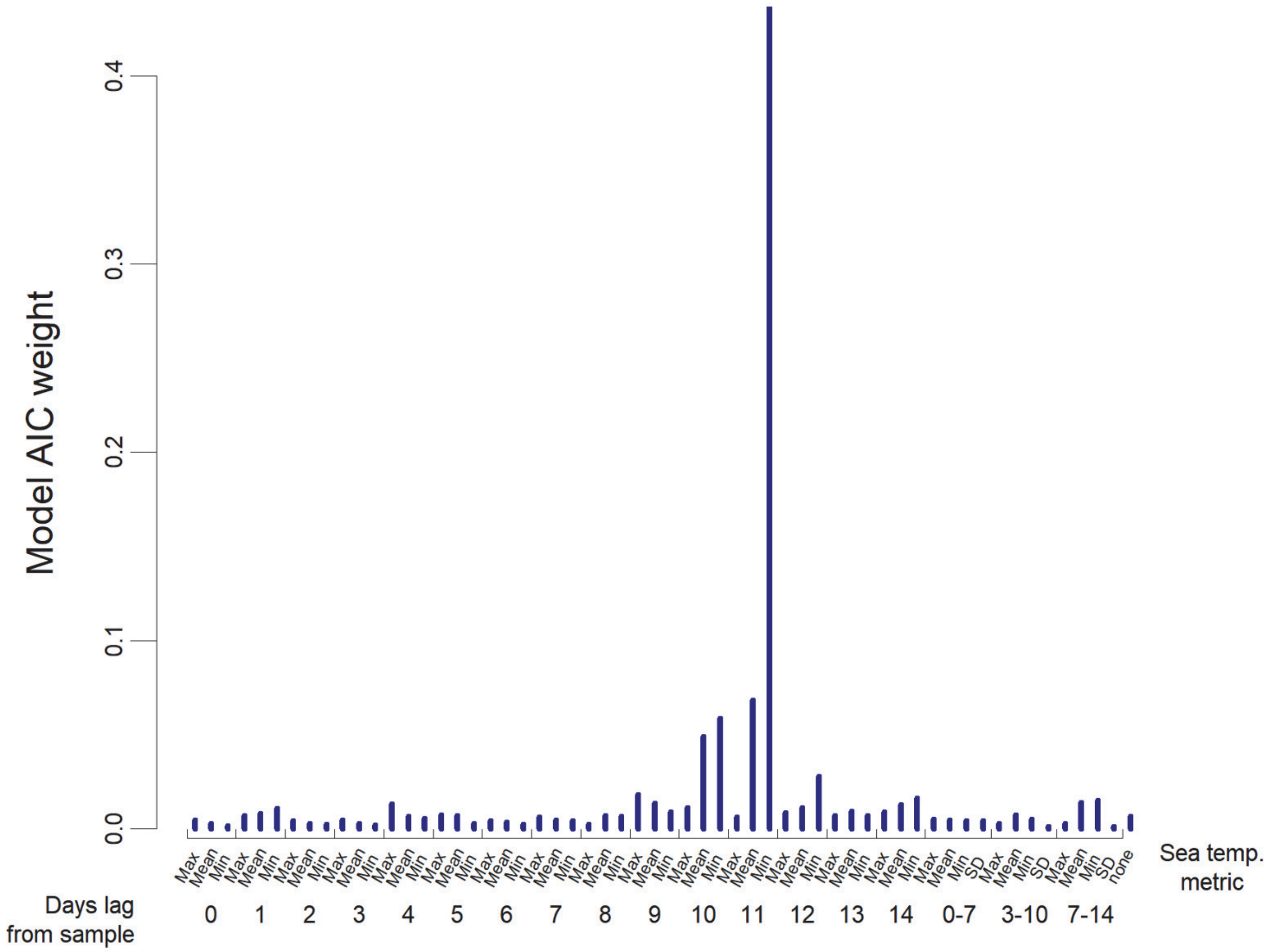

Supplement: Supplementary file 1 [file EAP-30-e02040-s001.pdf]
